# 

**Published:** 1886-05

**Authors:** 


### THE AMERICAN JOURNAL —OF— DENTAL SCIENCE,

—EDITED BY—

F. J. S. GORGAS, M. D., D. D. S.

---

VOLUME XX—THIRD SERIES.

---

1886-87.

SURGEON GEN'L'S OFFICE
LIBRARY
115694

BALTIMORE:
SNOWDEN & COWMAN,
LONDON:
TRUBNER & CO., 60 PATERNOSTER ROW.
1887.

### Index to Volume XX.---Third Series.

A Disinfectant, - - - - - - 480
A Porcelain Filling, - - - - - - 286
A Peculiar Case of Neuralgia of the Trigeminus, - 131
A Logical Inference, - - - - - - 411
A Defense of English Dentistry, - - . - 42
A New Substance, - - - - - - 378
A Doctor's Luck, - - - - - - 47
A Singular and Interesting Case, - - - 570
A Remarkable Case, - - - - 95
A Dear Kiss, - - - - - - - 528
A Visit to Foreign Dental Schools, - - - 110
A Curious Christmas Box, - - - - 384
A Criticism on the Herbst Method of Filling Teeth, 399
American Dentists Abroad, - - - - 43
American Dental Association, - - - 89, 185
Artificial Ivory, - - - - - - - 47
Advice to Those who Wear Artificial Teeth, - 127
All Porcelain Crowns and Bridge-Work, - - 188
Aluminium Bronze in Medicine and Dentistry, - 237
Anæsthesia vs. Asphyxia, - - - - - 289
An International Dental Congress, - - - 472
Arsenic in Destroying Pulps of Teeth, - - - 39
Annual Commencement of University of Maryland,
Dental Department, - - 424, 473, 520
Accuracy as a Condition of Scientific Progress, - 350
Atmospheric Purification, - - - - - 140
Allport, W. W., M. D., D. D. S., - - - 399
Are Micro-Organisms Necessary to Pus Formation, 543
Bone, Reproduction of, - - - - - 73
Boric Acid and Affections of the Mouth, - - 238
Bridge-Work, - - - - - - 280
Beadles, E. P., D. D. S., . - - 385, 571
Bennett, A. G., D. D. S., - - - - - 12
Bellamy, Dr. Fred. A., - - - - - - 411

BIBLIOGRAPHICAL.

Diseases of Digestive Organs in Infancy & Childhood, 139
Facial Prosthesis, - - - - - - 140
The Therapeutics of High Temperature in Young Children, - - - - - 140
Reproduction of Tissue by Skin Grafting, - 140
Atmospheric Purification, - - - - 140
Address before Alumni Association of Medical and Surgical Department of University of Michigan, 140
The American System of Dentistry, Vol. I, II, 236, 573
A Practical Treatise on Mechanical Dentistry, - 334
Index to the Periodical Literature of Dental Science and Art, - - - - - 335
Transactions of American Dental Association, - 474
Caulk's Dental Annual for 1886 87, - - 474
The Dental Office and Laboratory, - - 474
The Dental Review, - - - - 474
James Vick's Floral Guide for 1887, - - 474
The Western Dental Journal, - - - 475
Cocaine in Tooth Extraction, - - - - 31
Cormack, Edw. A., L. D. S., Etc., - - - 49
Caries of Left Side of Inferior Maxilla, - - - 92
Care and Treatment of Children's Teeth, - - 179
Chloride of Iron, Effects on the Teeth of, - - 190
Chilcott, L. S., D. D. S., - - - - 179
Chicago College of Dental Surgery, - - - 572
Convulsions, Etc., - - - - - 240
Catching, B. H., D. D. S., - - - - 261
Cleft Palate, Method of Operating for, - - 335
Chemistry, - - - - - - 366
Cancrum Oris, - - - - - 414
Chloral Hydrate as a Vesicant, - - - - 527
Courtesy Among Dentists at Professional Meetings, 550
Diagnosis—Its Importance, - - - - 21
Dixon, F. M., D. D. S., - - - - - 1
Discoloration of Gold Fillings, - - - - 44
Danger from the Bicycle, - - - - 45
Death from Chloroform, - - - - 118
Distinguished Honor, - - - - - 142

Disagreeable Accident Connected with the Extraction of a Tooth, - - - - - - 187
Destructive Energy of Tincture of Chloride of Iron on the Teeth, - - - - - 190
Dental Jurisprudence, - - - - 228, 508
Dental Students, - - - - - 331
Dodge, J. Smith, M. D., D. D. S., - - - 350
Drumine—The New Local Anæsthetic, - - 405
Dentists Damaged by the Earthquake at Charleston, S. C. 432
Disinfectant, A, - - - - - - 480
Dentistry a Specialty of Medicine, - - - 499
Ehinger, C. E., M. D., - - - - 327
Effects of Chloride of Iron on the Teeth, - - 190
Evolution in Pathology, - - - - 565
Food Accessories—Their Influence on Digestion, - 37
Final Results of Operation for Cancer of the Lip, - 143
Fun Among the Doctors, - - - - 144
Farrar, J. N., M. D., D. D. S., - - - 104, 550
Fripp, John Trude, L. D. S., - - - - 145
Fatal Result of Peroxide of Hydrogen Injections, - 425
Filling Proximal Cavities, - - - - 427
Francis, C. E., D. D. S., - - - - 443
First Colored Dental School, - - - - 523
Genese, D., D. D. S., - - - - - 506
Gold Solder, - - - - - - 48
Georgia State Dental Board, - - - - 90
Grady, Richard, D. D. S., - - - 129, 228, 508
Grind It Off, - - - - - - - 576
Howard, F. E., M. D., - - - - - - 120
Hamecher, H., D. D. S., - - - - 131
How to Become Thin, - - - - - 141
Hodgkin, James B., D. D. S., - - - 157
Herbst, Dr. William, - - - - - - 233
Haskell, Dr. L. P., - - - - - - 280
History of Dentistry, - - - - - - 571
Hayes, Dr. S. J., - - - - - 289
Hydronaphthol—A New Antiseptic, - - - 382
How to Become a Good Dentist, - - - 426
Hardman, J., D. D. S., - - - - - 448

Humphreys, John, L. D. S., - - - 458
Hopkinson, B. M., M. D., D. D. S., - - - 499
International Medical Congress, - - 513, 525, 538
Improved Method for Operating for Cleft Palate, - 335
Intimacy Breeds Contempt, - - - 95
Irregularities of the Teeth and their Treatment, - 120
Implantation of Teeth, and Pericemental Life, - 264
Illinois State Dental Society, - - - - 569
Kurtz, Joseph, M. D., - - - - 73
Kava, Kawa, Kava-Kava—A Powerful Anæsthetic, and Spinal Depressant, - - - 372
Louisiana State Dental Association, - - - 423
Local Remedy for Neuralgia, - - - 480
Mucous Membrane of the Mouth, - - - 49
Maryland Dental Law, - - - - 129
Medical Education for Dentists, and Dental Education for Physicians, - - - - 261
Much or Little Water in Vulcanizing, - - - 273
Mouth-Breathing, - - - - - 377
Moody, J. D., D. D. S , - - - - 337
Miller, W. D., D. D. S., - - - - 390
Marshall, John S., M. D., - - - - 408
Man's Lost Incisors, - - - - - 458
Mirror-Topped Syphon Tongue-Holder, - - 506
Missouri State Dental Society, - - - 570
National Association of Dental Faculties, - - 175
Notes on the Decay of Human Teeth, - - 390
New Remedies, - - - - - - 23
Neuralgia of the Trigeminus, - - - 131
Nelson, Dr. D. T., - - - - - 394
New Method of Mounting an Artificial Denture, - 564
Œsophagotomy for Removal of an Artificial Denture, - 376
Occasional Serious Results of Pulp Treatment, - 1
Oxyphosphates and Temperature, - - - 44
On the Rotation Method—Construction of the First Gold Layers, - - - - - - 88
Odontological Society of Pennsylvania, - - 162
On the Treatment of Pyorrhœa Alveolaris, - - 276
Oxygen as a Therapeutic Agent, - - - - 327

Our Duty to our Profession, - - - - 385
Odontological Society of Great Britain, - - 415
Odonto-Chirurgical Society Proceedings, - - 381
OBITUARY.
Dr. Frank P. Abbott, - - - - 475
Dr. John G. Wayt, - - - - 525
Principles and Methods of Filling Teeth with Gold, 12
Poisoning from Amalgam, - - - - 65
Pocket Disease of the Alveolus, - - - 104
Prosthetic Dentistry, - - - - - - 157
Preparation of the Mouth for, and the Insertion of Artificial Teeth, - - - - - 145
Pyorrhœa Alveolaris, - - - - - 221
Porcelain Filling, - - - - - 286
Post-Graduate Study, - - - - - 337
Pin Hole Cavities and "Caries Interna," - - 378
Preventive Dentistry, - - - - - 379
Power of Operator over Patient under Influence of Nitrous Oxide Gas, - - - - - 381
Personal Cleanliness and Unpleasant Odors and Disease, 428
Physicians Extracting Teeth, - - - - 431
Protoplasmic Nutrition and Molecular Metamorphosis in the Dental Tissues, - - - - 433
Professional Courtesies in Dental Practice, - - 443
Period of Puberty and the Teeth, - - - 394
Repairing a Cracked Plate, - - - 47
Reproduction of Bone, - - - - - 73
Recent Progress in Dentistry, - - - 79, 97
Rollins, Dr. William Herbert, - - - - 79, 97
Requirements for Graduation in Medicine and Dentistry, in England and Scotland, - - - 283
Requirements of Teeth Extraction During Youth, - 448
Restoring a Bicuspid, - - - - - 478
Resorcin Inoculations in Phlegmon, - - 575
Remedy for Tired Eyes, - - - - - 479
Runyan's Method of Bridge-Work, - - 565
Southern Dental Association, - 135, 183, 193, 241, 296
Sharpening Corundum Wheels, - - - 48
Sedative Cough Mixture, - - - - 575
Source of Scarlet Fever, - - - - 137

Smoking, Prof. Huxley on, - - - - - 142
Snow, Dr. George B., - - - - - 273
Starr, Alfred R., M. D., D. D. S., - - - 221
Swallowing a Set of Artificial Teeth, - - 336
Steel, Charles L., M. D., D. D. S., - - - 366
Superior Wisdom Tooth Discharged from Nasal Passages, 408
Surgical Diseases of the Tongue, - - - 454
Smith, H. A., D. D. S., - - - - - 454
Something New in Making Rubber Plates, - - 91
The Point of Death, - - - - - 332
Thompson, Alton H., D. D. S., - - - 433
The Pasteur Methods, - - - - - 424
Treatment of Blind Abscesses, - - - - 562
The Most Popular Medicines, - - - 432
Taft, J., M. D., D. D. S., - - - - 124
The Treatment of Pulpless and Gaseous Teeth, - 124
Transplantation of Teeth, - - - - 559
Tuberculosis of the Buccal Mucous Membrane, - 94
The Treatment of Sick Headache, - - - 96
The General Structure of the Teeth, - - 529
University of Maryland, Dental Department, 232, 282, 424, 520
Wire Ligatures, - - - - - - 288
Warts, - - - - - - - 575
White of Egg in Obstinate Diarrhœa, - - - 46
Wallis, C. J. Boyd, L. D. S., - - - 21
Whatford, Dr. J. Henry, - - - - - 276
Younger, William J., M. D., - - - 264

### To Readers and Correspondents.

Communications intended for publication, and exchange journals and papers should be addressed to the Editor of the American Journal of Dental Science, 259 N. Eutaw St., Baltimore, Md. All letters relating to the business of the Journal, or containing cash remittances, to be directed to Messrs. Snowden & Cowman. Articles for publication should be received by the editor at least three weeks previous to the first month on which the number in which it is designed for them to appear, is issued.

### CONTENTS:

Oxyphosphates and Temperature ........ 44

Discoloration of Gold Fillings ........ 44

Danger from the Bicycle ........ 45

White of Egg in Obstinate Diarrhœa ........ 46

A Doctor's Luck ........ 47

Artificial Ivory ........ 47

Repairing a Cracked Plate ........ 47

Sharpening Corundum Wheels ........ 48

Gold Solder ........ 48

☞ The American Journal of Dental Science will contain fifty pages of reading matter in each number, making a volume of about

*Six Hundred* pages,

EXCLUSIVE OF ADVERTISEMENTS.

